# The mTORC1-autophagy pathway is a target for senescent cell elimination

**DOI:** 10.1007/s10522-019-09802-9

**Published:** 2019-02-23

**Authors:** Olena Kucheryavenko, Glyn Nelson, Thomas von Zglinicki, Viktor I. Korolchuk, Bernadette Carroll

**Affiliations:** 1 Institute for Cell and Molecular Biosciences, Newcastle University, Newcastle upon Tyne, NE4 5PL UK; 2 Present Address: The Federal Institute for Risk Assessment, 10589 Berlin, Germany

**Keywords:** mTOR, Senescence, DNA damage, Torin1, Ageing

## Abstract

Cellular senescence has recently been established as a key driver of organismal ageing. The state of senescence is controlled by extensive rewiring of signalling pathways, at the heart of which lies the mammalian Target of Rapamycin Complex I (mTORC1). Here we discuss recent publications aiming to establish the mechanisms by which mTORC1 drives the senescence program. In particular, we highlight our data indicating that mTORC1 can be used as a target for senescence cell elimination in vitro. Suppression of mTORC1 is known to extend lifespan of yeast, worms, flies and some mouse models and our proof-of-concept experiments suggest that it can also act by reducing senescent cell load in vivo.

## Introduction

Cellular senescence is a potent tumour suppressor mechanism that also plays an important role in wound healing and development. There is evidence, particularly in mouse models that it can however turn from protector to antagonist with increasing age when these metabolically active cells accumulate in tissues (de Magalhaes and Passos 2018). Replicative exhaustion, DNA damage, excessive stress and oncogene activation can all activate the senescence program, resulting in an irreversible exit from the cell cycle. Senescence is characterised by an increase in cell size, increased organelle content and a robust senescence-associated secretory phenotype (SASP). The accumulation of senescent cells within a number of tissues including lung (Birch et al. 2015), muscle (Sousa-Victor et al. 2014), liver (Ogrodnik et al. 2017) and bone (Farr et al. 2017) is associated with reduced tissue regeneration capacity, function and integrity. Moreover the inflammatory environment mediated by SASP has been intimately linked with driving gross tissue changes such as fibrosis (Schafer et al. 2018) and increased risk of cellular transformation via the so-called bystander effect (Schosserer et al. 2017; Rao and Jackson 2016). Seminal work from the Mayo Clinic demonstrated that clearance of p16/Ink4a positive cells (a marker of senescence) can improve age-related pathologies and lengthen healthy lifespan in wild-type and progeroid mice (Baker et al. 2011; Baker et al. 2016). Others have further demonstrated the health benefits of removing senescent cells in mouse models, including an improvement in post-trauma tissue regeneration and alleviating chemotherapy-associated fatigue (Jeon et al. 2017; Demaria et al. 2017). This latter report further described that T cell p16/Ink4a expression in humans correlates with severity of chemotherapy drug toxicity indicating their clearance could be therapeutically beneficial. Due to the economic, social and medical burden of ageing in the Western world, the need to find interventions to improve healthspan is fundamentally important. Understanding more about the mechanisms driving and maintaining senescence and senescence-associated phenotypes may support the identification of targeted, safe interventions to meet this ultimate goal.

The central role of the mammalian target of rapamycin complex 1 (mTORC1) in driving senescence-associated phenotypes including SASP and increased mitochondrial content has been comprehensively established (Korolchuk et al. 2017; Lopez-Otin et al. 2013; Correia-Melo. et al. 2018). Further to this, we recently demonstrated that mTORC1 dependency may represent a targetable vulnerability in senescent cells that could be used to eliminate them (Carroll et al. 2017). mTORC1 is a master regulator of cell growth, the activity of which is tightly controlled by the balance between mitogenic and stress signals. Some of the most potent activators of mTORC1 are growth factors and amino acids which work at least in part to control the localisation and thus activation of the mTORC1 complex on the lysosome/late endosome surface. It is on the lysosomal surface that mTORC1 can ‘sense’ changes in amino acids and growth factors via a huge number of regulatory proteins and complexes including the Rag-Ragulator complexes and Tuberous Sclerosis Complex (TSC) (Carroll and Dunlop 2017; Wolfson and Sabatini 2017; Ben-Sahra and Manning 2017). In its active form, mTORC1 drives protein translation, lipid and nucleotide synthesis as well as inhibiting the catabolic process of autophagy to support cell growth and metabolism. During periods of limiting nutrient availability, mTORC1 is switched off and autophagy activity increases to sequester cytoplasmic material and deliver it to the lysosomes for degradation. Liberation of the resulting free amino acids, lipids and carbohydrates from the lysosome supports cell survival (Carroll et al. 2015).

 Senescent cells undergo dramatic rewiring of these pro-growth and scavenging mechanisms that drive their increased metabolism and survival (Carroll et al. 2017; Narita et al. 2011). In particular we recently demonstrated that mTORC1 activity is resistant to starvation of amino acids and growth factors in senescence which prevents starvation-induced activation of autophagy (Carroll et al. 2017). Mechanistically, the basal expression of autophagy and lysosome proteins is grossly elevated in senescence and this may contribute to increased levels of intracellular amino acids which activate mTORC1 and render it insensitive to the availability of exogenous nutrients. Thus senescence results in a unique re-equilibrium between mTORC1 and autophagy (Carroll et al. 2017; Carroll and Korolchuk 2018). We identified that this can be targeted to kill senescent cells; treatment with lysosome inhibitors or Torin1 in the absence of exogenous nutrients selectively causes senescent cell death *in vitro* (Carroll et al. 2017).

## Methods

Animal procedures were performed according to protocols approved by the Home Office (Personal Licence 60/4542 (OK)) and animal health was monitored according to FELASA guidelines. Male immunodeficient NSG mice were purchased from Charles River UK breeding facility. Mice (13-17 weeks old) were randomly assigned to groups at the commencement of the treatment mice. Mice in both groups had ad libitum access to standard chow and water. Mice were injected intraperitoneally with a solution of 20 mg/kg Torin1 (Tocris) or vehicle every other day for 7 days. Torin1 solution was prepared in 100% N-methyl-2-pyrrolidone and diluted with sterile PEG400 and water for injections at the ratio of 1:2:2 immediately prior to injection. Liver tissue was harvested in 10% solution of paraformaldehyde (PFA) and 24 h later were paraffin embedded. Three independent animal experiments were quantified.

Paraffin sections (3 μm) were cut and subjected to immunofluorescence and in situ hybridisation staining as described previously (da Silva et al. 2019) using primary rabbit anti-γH2AX (CST #9718, 1:250) and Cy3-labelled telomere specific (C3TA2)3 peptide nucleic acid (PNA) probe (4 ng μl^−1^, Panagene) using denaturation at 80 °C and hybridization for 2 h at room temperature in the dark. Confocal z stack images were collected on an SP8 Confocal Microscope (Leica) using a 63X Plan-Apo/1.4 NA Oil objective at Nyquist sampling density. Images were analysed with ImageJ software (https://imagej.nih.gov/ij/). γH2A.X- and telomere overlap, and γH2A.X foci were manually counted. A minimum of 100 nuclei were counted per animal. Statistical analysis (T tests between animal means) was performed with Sigmaplot software (v 13, Systat Software Inc.).

## Results and Discussion

**Fig. 1:**
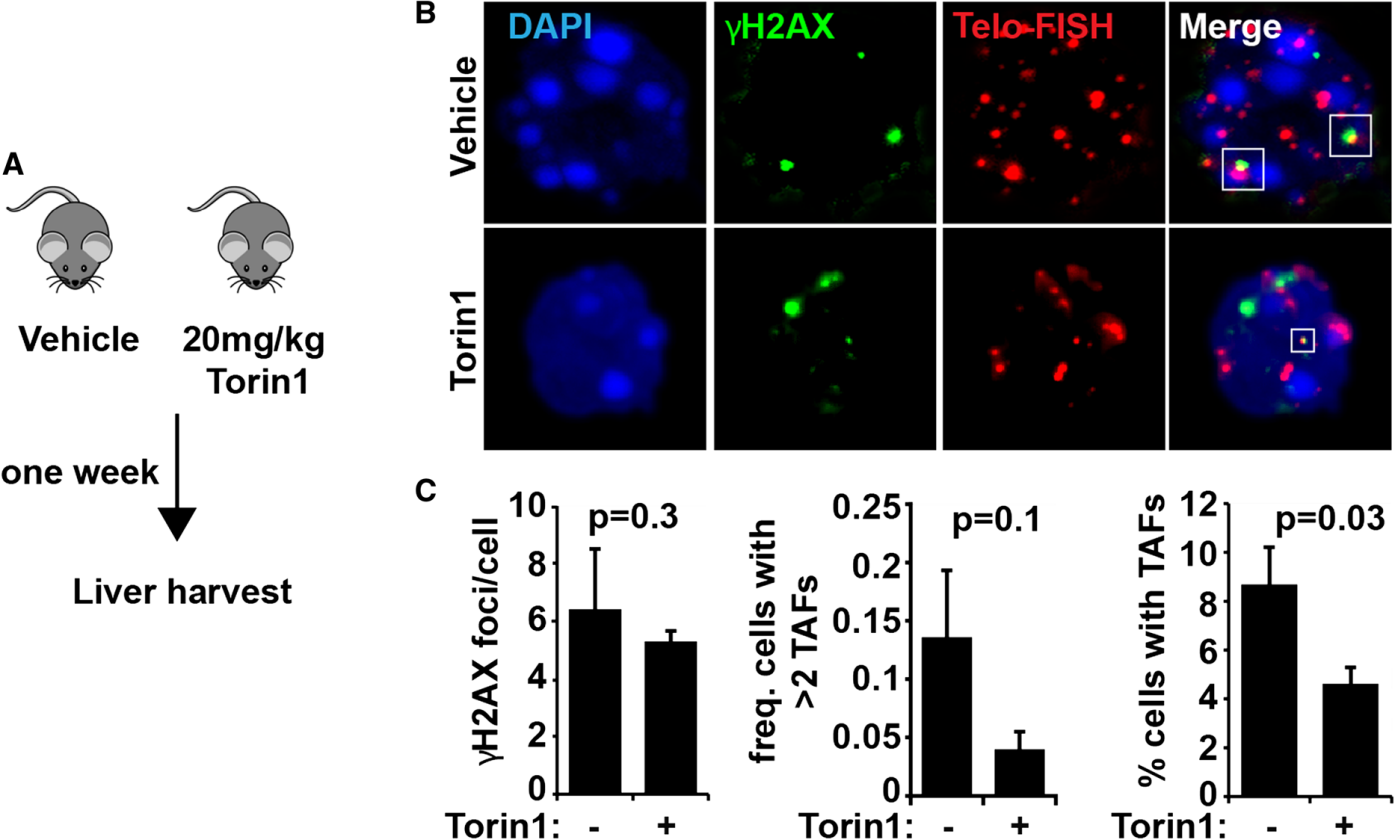
Mice treated with Torin1 show reduced markers of senescence. **a** Diagram depicting the Torin1 treatment protocol. **b**, **c** Mouse liver samples were analysed for markers of DNA damage (γH2AX) and senescence-associated telomere-associated DNA damage foci (see co-localisation of telo-FISH probe and γH2AX)

Our proof-of-principle study demonstrates that targeting persistent mTORC1 in vivo reduced the number of hepatocytes harbouring a key marker of senescence, telomere-associated DNA damage foci (TAFs) as measured by co-localisation of a telomere probe and γH2AX (Fig. 1a–c) (Hewitt et al. 2012; Fumagalli et al. 2012). Indeed, both the percentage of cells with TAFs, and the percentage of cells with more than two TAFs decreased after short term (7 day) administration of the mTORC1 inhibitor, Torin1, while the overall number of γH2AX DNA damage foci did not decrease which indicates that the effect of Torin1 on senescent cells is not merely a result of reduced DNA damage (Fig. 1c).

It is important to note that the Torin1 treatment was performed in NOD Scid Gamma (NSG) mice that lack mature T cells, B cells and natural killer cells and are deficient in innate immunity and in multiple cytokine pathways. These mice accumulate senescent hepatocytes much faster than wild-type mice. We have shown previously that dietary restriction (60% of ad libitum food intake) for 3 months reduced senescent cell frequency in livers of wild-type mice below starting values (Ogrodnik et al. 2017), while senescent hepatocyte frequency remained constant in NGS mice (da Silva et al. 2019). It is possible that mTORC1-suppressing interventions like dietary restriction or Torin1 simply diminish the senescent phenotype without ablating the cells itself, and this is what many have seen in vitro (Correia-Melo et al. 2016; Demidenko et al. 2009). However, the reduction in senescent cell frequency by dietary restriction in wild-type mice was still evident at least 3 months after return to ad libitum feeding (Ogrodnik et al. 2017). Moreover, in vitro, the combination of starvation and mTORC1 inhibition acted synergistically to eliminate senescent cells (Carroll et al. 2017). Thus, it is possible that the reduction in senescent markers shown in Fig. 1 was due to senescent hepatocyte elimination caused by cell death rather than resulting from phenotypic suppression or reduced induction/accumulation. While these findings are preliminary and require robust validation with additional senescence markers, they support our in vitro findings and considering that inhibition of mTORC1 in vivo is well tolerated, it warrants further investigation and could represent a potentially powerful intervention. While it is an exciting prospect that we could one day identify simple interventions to promote longer, healthy human lifespan, future studies will have to investigate the repercussions of inducing senescent cell death on tissue integrity, particularly in tissues with low regeneration capacity. Furthermore, understanding the mechanisms via which cell death occurs (i.e. apoptosis, autophagy, necrosis) will be an important consideration due to the potential damage that excessive inflammatory and toxic factors could cause to neighbouring tissue.

These are still early days in our quest to find senolytic agents but mTORC1 may provide an ideal target. Not only has its inhibition via multiple interventions including rapamycin and calorie restriction been reproducibly shown to slow the ageing process in a wide range of model organisms including yeast, worms, flies and many mouse models (Weichhart 2018), but our new data indicate that mechanistically this could at least in part be due to its senolytic activity.

## References

[CR1] Baker DJ (2011). Clearance of p16Ink4a-positive senescent cells delays ageing-associated disorders. Nature.

[CR2] Baker DJ (2016). Naturally occurring p16(Ink4a)-positive cells shorten healthy lifespan. Nature.

[CR3] Ben-Sahra I, Manning BD (2017). mTORC1 signaling and the metabolic control of cell growth. Curr Opin Cell Biol.

[CR4] Birch J (2015). DNA damage response at telomeres contributes to lung aging and chronic obstructive pulmonary disease. Am J Physiol Lung Cell Mol Physiol.

[CR5] Carroll B, Dunlop EA (2017). The lysosome: a crucial hub for AMPK and mTORC1 signalling. Biochem J.

[CR6] Carroll B, Korolchuk VI (2018). Nutrient sensing, growth and senescence. FEBS J.

[CR7] Carroll B, Korolchuk VI, Sarkar S (2015). Amino acids and autophagy: cross-talk and co-operation to control cellular homeostasis. Amino Acids.

[CR8] Carroll B (2017). Persistent mTORC1 signaling in cell senescence results from defects in amino acid and growth factor sensing. J Cell Biol.

[CR9] Correia-Melo C (2016). Mitochondria are required for pro-ageing features of the senescent phenotype. EMBO J.

[CR10] Correia-Melo C (2018). Rapamycin improves healthspan but not inflammaging in nfkappab1(-/-) mice. Aging Cell.

[CR11] da Silva PF, Kucheryavenko O, Gilbert J, Miwa S, Cameron K, Ishaq A, Saretzki G, Nagaraja-Grellscheid S, Nelson G, von Zglinicki T (2019). The bystander effect contributions to the accumulation of senescent cells in vivo. Aging Cell.

[CR12] de Magalhaes JP, Passos JF (2018). Stress, cell senescence and organismal ageing. Mech Ageing Dev.

[CR13] Demaria M (2017). Cellular senescence promotes adverse effects of chemotherapy and cancer relapse. Cancer Discov.

[CR14] Demidenko ZN (2009). Rapamycin decelerates cellular senescence. Cell Cycle.

[CR15] Farr JN (2017). Targeting cellular senescence prevents age-related bone loss in mice. Nat Med.

[CR16] Fumagalli M (2012). Telomeric DNA damage is irreparable and causes persistent DNA-damage-response activation. Nat Cell Biol.

[CR17] Hewitt G (2012). Telomeres are favoured targets of a persistent DNA damage response in ageing and stress-induced senescence. Nat Commun.

[CR18] Jeon OH (2017). Local clearance of senescent cells attenuates the development of post-traumatic osteoarthritis and creates a pro-regenerative environment. Nat Med.

[CR19] Korolchuk VI, Miwa S, Carroll B, von Zglinicki T (2017). Mitochondria in cell senescence: is mitophagy the weakest link?. Ebiomedicine.

[CR20] Lopez-Otin C, Blasco MA, Partridge L, Serrano M, Kroemer G (2013). The hallmarks of aging. Cell.

[CR21] Narita M (2011). Spatial coupling of mTOR and autophagy augments secretory phenotypes. Science.

[CR22] Ogrodnik M (2017). Cellular senescence drives age-dependent hepatic steatosis. Nat Commun.

[CR23] Rao SG, Jackson JG (2016). SASP: tumor suppressor or promoter? yes!. Trends Cancer.

[CR24] Schafer MJ, Haak AJ, Tschumperlin DJ, LeBrasseur NK (2018). Targeting senescent cells in fibrosis: pathology, paradox, and practical considerations. Curr Rheumatol Rep.

[CR25] Schosserer M, Grillari J, Breitenbach M (2017). The dual role of cellular senescence in developing tumors and their response to cancer therapy. Front Oncol.

[CR26] Sousa-Victor P (2014). Geriatric muscle stem cells switch reversible quiescence into senescence. Nature.

[CR27] Weichhart T (2018). mTOR as regulator of lifespan, aging, and cellular senescence: a mini-review. Gerontology.

[CR28] Wolfson RL, Sabatini DM (2017). The dawn of the age of amino acid sensors for the mTORC1 pathway. Cell Metab.

